# ILC-OPI: impulsive lifestyle counselling versus cognitive behavioral therapy to improve retention of patients with opioid use disorders and externalizing behavior: study protocol for a multicenter, randomized, controlled, superiority trial

**DOI:** 10.1186/s12888-021-03182-6

**Published:** 2021-04-07

**Authors:** Morten Hesse, Birgitte Thylstrup, Sidsel Helena Karsberg, Michael Mulbjerg Pedersen, Mads Uffe Pedersen

**Affiliations:** Centre for Alcohol and Drug Research, Bartholins Allé 10, 8000 Aarhus C, Denmark

**Keywords:** Opioid use disorder, Opioid agonist treatment, Externalizing disorders, Psychoeducation, Retention, Co-morbidity, Impulsive lifestyle Counselling

## Abstract

**Background:**

Substance use disorders show a high comorbidity with externalizing behavior difficulties, creating treatment challenges, including difficulties with compliance, a high risk of conflict, and a high rate of offending post-treatment. Compared with people with other substance use disorders those with opioid use disorders have the highest risk of criminal activity, but studies on the evidence base for psychosocial treatment in opioid agonist treatment (OAT) are scarce. The Impulsive Lifestyle Counselling (ILC) program may be associated with better retention and outcomes among difficult-to-treat patients with this comorbidity.

**Methods:**

The study is a multicenter, randomized, controlled, superiority clinical trial. Participants will be a total of 137 hard-to-treat individuals enrolled in opioid agonist treatment (OAT). Participants will be randomized to either a standard treatment (14 sessions of individual manual-based cognitive behavioral therapy and motivational interviewing (MOVE-I)) or six sessions of ILC followed by nine sessions of MOVE-I. All participants will receive personalized text reminders prior to each session and vouchers for attendance, as well as medication as needed. The primary outcome is retention in treatment. Secondary measures include severity of drug use and days of criminal offending for profit three and nine months post-randomization. A secondary aim is, through a case-control study, to investigate whether participants in the trial differ from patients receiving treatment as usual in municipalities where ILC and MOVE-I have not been implemented in OAT. This will be done by comparing number of offences leading to conviction 12 months post-randomization recorded in the national criminal justice register and number of emergency room contacts 12 months post-randomization recorded in the national hospital register.

**Discussion:**

This is the first randomized, controlled clinical trial in OAT to test the effectiveness of ILC against a standardized comparison with structural elements to increase the likelihood of exposure to the elements of treatment. Results obtained from this study may have important clinical, social, and economic implications for publicly funded treatment of opioid use disorder.

**Trial registration:**

ISRCTN, ISRCTN19554367, registered on 04/09/2020.

## Background

Substance use disorders are increasingly considered a part of the externalizing spectrum of psychopathology. Under the P-model of psychopathology, externalizing and internalizing spectrum disorders constitute dimensions of psychopathology in disorders associated with behavioral difficulties and emotional difficulties, respectively [1, 2]. Externalizing spectrum disorders refer to disorders that are characterized by impulsivity and aggression, such as antisocial personality disorder, intermittent explosive disorder, and hyperactivity, and internalizing spectrum disorders refer to disorders that are characterized by internal suffering, such as anxiety, depression, eating disorders, and self-harm. Latent class models indicate that classes characterized by high levels of externalizing difficulties have the highest degree of comorbidity with substance use disorders in the general population [1], as well as in other groups such as prison inmates [3].

In treatment services for substance use disorders, internalizing psychopathology is associated with higher levels of distress, whereas externalizing psychopathology is associated with complicated treatment processes, such as difficulties with other residents in rehabilitation facilities [4] and dropout from treatment [5]. However, the relationship is highly variable [6] and may especially be present when the patient has little to gain from being in treatment beyond his or her intrinsic motivation to change substance use behavior [7]. Furthermore, several studies show that externalizing psychopathology correlates with (re-)offending after treatment for substance use disorders [8, 9] and failure to obtain employment [9]. By contrast, we are not aware of strong evidence that distress by itself is associated with poor outcomes in treatment for substance use disorders.

Compared with patients with other substance use disorders, people with opioid use disorders have the highest risk of criminal activity, psychiatric comorbidity, poly-substance use, infections, such as HIV and hepatitis C [10, 11], as well as excess mortality [12, 13] and a high degree of somatic comorbidity [14]. However, this is primarily due to the fact that opioid use disorders represent the most severe end of the substance use disorder spectrum, and while opioid use disorder is associated with antisocial personality traits, this is mostly as part of its overall relationship with a general propensity for substance use disorders [15]. Once opioid use disorder is established, however, criminal offending may be reduced by the provision of medication assisted treatment [16].

The dual burden of using a drug with particularly heavy consequences and externalizing behavior problems may put some patients at very high risk for a range of adverse outcomes. A considerable barrier to benefitting from treatment is staying in contact with the system for a sufficient time; around 30% of patients drop out of psychosocial treatment across types of substances and treatment, and dropout rates are even higher for more disadvantaged patients and treatments that demand time and concentration [17, 18]. Furthermore, a growing body of evidence suggests that patients with externalizing and criminal behavior are particularly hard to retain in treatment and offer relevant help but that improved retention and treatment effects may be associated with substantially lower costs of crime [19, 20]. Thus, improving retention and outcomes for people with externalizing disorders who enroll in OAT is of crucial importance.

One way to improve retention and outcomes is the use of contingency management (CM) in the form of voucher-based reinforcement therapy [21]. However, the evidence base for specially tailored programs to serve the needs of people with opioid use disorder who are high on externalizing problems is extremely limited. A recent narrative review based on a small handful of studies indicated that cognitive behavioral therapy and CM, or milieu therapy alone or in combination with other interventions, have not been differentially effective for patients with opioid use disorder and antisocial personality disorder [22].

One program that has been tested in a single multicenter trial is the Impulsive Lifestyle Counselling (ILC) program [23, 24], which was developed as an add-on to treatment for substance use disorders with co-morbid antisocial personality disorder in outpatient settings. ILC is a six-session psycho-educative program delivered face-to-face that focuses on increasing self-understanding and on linking impulsive and destructive behaviors to their immediate negative consequences, supporting motivation for change (see Table 1). The trial showed that random assignment to ILC was associated with a reduction in dropout and substance use behaviors [23, 24]. However, the comparison treatment was not standardized and was based on a “treatment as usual” approach. It is well-documented that the less defined and structured the comparison condition is, the higher the estimated efficacy of treatment [25]. Another limitation was that the participants in the trial were very heterogeneous, representing both opioid, alcohol, cannabis, and stimulant users [23, 24]. An advantage to such a heterogeneous sample is that it prioritizes external validity [26, 27], as treatment units for people with substance use disorders often serve people with poly-substance use. However, this comes at a cost because such trials are unable to inform clinicians and administrators about the value of the intervention for more specific target groups.

**Table 1:** Headings of the sessions of Impulsive Lifestyle Counselling

Session	Content
1. Assessment	AdultMap assessment of strengths and challenges
2. Treatment plant	Collaborative development of a treatment plan, including the ILC program
3. Introduction	Dreams, goals, and four areas of an impulsive lifestyle (self-indulgence, breaking rules, interpersonal intrusiveness, and irresponsibility).
4. The TAC-model: Triggers, Reactions, Consequences	Learn about the connections between triggers, behaviors and consequences and begin to apply the model.
5. Streetwise pride and self-worth	Understand the pride associated with antisocial behavior, deceptiveness and crime and think about alternative sources of pride and self-worth
6. Values	Identify values that support or under-mine change in externalizing behavior.

While OAT with methadone, buprenorphine, or buprenorphine plus naloxone has been shown to be effective at reducing use of illicit opioids, preventing drug-related deaths, and reducing overall healthcare costs of opioid use disorder [28], available evidence-based treatment interventions that target externalizing behavior are scarce, at least in adult patients. In summary, there is a lack of research on how the ILC program may work for patients with opioid use disorder and externalizing behavior who are enrolled in OAT. Given the global opioid epidemic that has led to increased fast dissemination of OAT [29], and further pressure to increase access even more [30], the current study has only become more necessary.

### Study objectives

The main aim of the study is to assess whether the extra effort required to deliver a different treatment content in OAT helps retain patients in treatment.

The ILC for Opioid Users trial (ILC-OPI trial) evaluates the feasibility, acceptability, and effectiveness of a combination of ILC and integrated cognitive behavior therapy and motivational interviewing (MOVE-I) versus MOVE-I in OAT in four community-based public treatment centers in Denmark.

#### Main objectives

1. To test whether the combination of ILC/MOVE-I will improve treatment retention compared with MOVE-I for patients with externalizing behavior enrolled in OAT.
2. To use case controls from the Register of Drug Abusers in Treatment to examine if ILC and MOVE-I are superior to treatment as usual in municipalities where ILC and MOVE-I have not been implemented in OAT, as measured by number of offences leading to conviction and number of emergency room contacts 12 months post-randomization.

### Methods/design

The study is a multicenter, randomized, controlled, superiority trial with two treatment conditions: ILC and MOVE-I.

### Study setting and recruitment

Participants (*N* = 137) will be recruited from OAT in four outpatient municipal treatment centers situated in four Danish cities: Herning, Randers, Aarhus, and Aabenraa. Both current patients and new admissions will be invited to participate. The study is situated at the Centre for Alcohol and Drug Research, Aarhus University.

### Eligibility criteria

Inclusion criteria are as follows: 1) seeking or currently receiving treatment for opioid use disorder at one of the participating sites; 2) over 18 years of age; 3) able to attend the evaluation and treatment sessions 4) score of three or above on the YouthMap12 Externalizing difficulties subscale [1].

Patients will be excluded if they have a severe mental disorder (e.g. current psychosis), cognitive difficulties, or severe aggressive and chaotic behavior that would preclude their participation in the counselling sessions.

Social workers and/or nurses at each participating site will assess whether an individual is eligible for study participation according to the eligibility criteria during the first in-person contact with the participating treatment sites, or among the patients already enrolled in OAT at the sites. If the individual is deemed eligible, the same employee will inform the individual about the trial verbally, and provide a participant information sheet. If the individual gives informed consent for study participation, the employee will collect the necessary information for randomization in an online questionnaire, which will be forwarded to the Centre for Alcohol and Drug Research, Aarhus University via an anonymous and secure email. The research team will ensure the confidentiality of this data by keeping the online questionnaire data and consent forms stored on secured servers. After randomization, the study participant will be assigned to one of the two treatment conditions that are tested in the study. Participants and clinicians cannot be blinded to the conditions that the participants will be randomized to, but the persons who will conduct the assessments at the follow-ups will be blinded.

### Clinicians and trial interventions

Clinicians in this trial will be employed by the participating sites as treatment counsellors and will receive training in the two conditions. The clinicians will all be taken from the existing staff at the clinics. During the trial, the counsellors will meet to discuss the implementation of the two conditions and will be supervised. In addition, after each session, the clinician will send in an electronic record to confirm that the patient has attended the session and will have the opportunity to contact the research team about any questions that have emerged. All assessments, as well as treatment planning and treatment sessions in the two conditions will be audio recorded. Randomly selected recordings that involve all counsellors who conduct each of the tested interventions will be coded, using the SBIRT coding system [31] to ensure intervention fidelity and integrity.

All participants will receive text reminders prior to sessions and a voucher worth DKK 200 for every two sessions attended (equal to approximately US$ 30). A session typically lasts around 30–45 min, depending on the client’s patience and ability to maintain concentration. When clients miss a single session without noticing the counsellor, they lose one voucher. Clients who miss three consecutive sessions without notice are discharged from the project, but not necessarily from treatment, and will still be included in the intention-to-treat analysis.

#### ILC

ILC is a brief, highly structured psychoeducational program targeting impulsive and destructive behaviors. The original manual is available in English (DOI: 10.13140/RG.2.2.27332.01927). In the two first sessions, the focus is on assessment and establishing treatment goals. Following these two sessions, the patient proceeds to six pre-defined sessions, each covering a different topic (see Table 1). Following ILC, participants will receive six of the sessions of MOVE-I. The content of these six sessions will depend on what themes in the MOVE-I manual are deemed relevant at this stage in treatment by the counsellor and the participant.

#### Move-i

MOVE-I [32] will include 14 sessions of integrated motivational interviewing and cognitive behavioral therapy. In the two first sessions, the focus is on assessment and establishing treatment goals, and in the following three sessions, the focus is on handling risk situations and preventing relapse. These five initial sessions are followed by nine theme-based sessions. The succession of these themes will depend on individual treatment goals and needs based on a dialogue between the clinician and the client.

#### Continued counselling

After the initial 14 sessions in the two conditions, participants in both arms will continue treatment as needed following the standard of care in the clinic.

#### Pharmacotherapy

Participants in both study arms will receive OAT under the Danish legislation and public guidelines for OAT [33], in addition to access to case management according to the Danish law of Social Services, § 101. Although heroin-assisted treatment is available to patients in Denmark, this treatment is not available at sites participating in this trial, and, therefore, participants who wish to initiate heroin-assisted treatment will have to be discharged from the trial. Pharmacotherapy for other substance use disorders will be delivered as treatment as usual by the physician affiliated with the treatment site and will be based on treatment needs and patient preference, regardless of randomization assignment. Treatment for other mental health problems is available to all participants via the public health insurance in Denmark through either their general practitioner or specialist services within the health care services.

### Outcomes

The primary outcome measure is retention in OAT. Retention in treatment will be calculated starting on the date of randomization and until the patient is discharged or the trial ends. Patients who are registered as having completed a successful course of treatment will be considered censored at the date of discharge, as was the case in the pilot trial [23]. Any reason for discharge, other than completed treatment, will be considered dropout, even if discharge is on patient request. In general, discharge occurs when the patient no longer receive any services. Note that practices may vary slightly between sites in terms of their practices when discharging patients. As researchers we are legally and practically unable to influence these decisions. None of the participating sites discontinue OAT against patients’ wishes, neither because of substance use, nor because of failure to attend treatment. Only the present episode is considered in this trial (i.e., the episode that is initiated or ongoing at the time of randomization).

The secondary outcomes are 1) substance use other than prescribed medications (AdultMAP) [1]; 2) self-reported days in the last month with offences for profit (AdultMAP); 3) substance use and everyday functioning (concentration, planning, keeping appointments, sleeping, eating habits, cleaning etc.) in the last month (AdultMap) and last week (WOM); 4) social interaction with individuals (family, friends, acquaintances) with non-criminal behavior and/or with no substance abuse in the last month (AdultMap); 5) employment/educational activities in the last month (AdultMap).

### Other measures

All patients in Denmark are registered by their personal identification number. The data collected the individual participants in the trial conditions will be compared using data on the same individuals from the Danish Register of Drug Users in Treatment; the Danish National Patient Register; the Danish Registry for Causes of Death; the Psychiatric Central Research Register; and the Danish Central Crime Register. The specific measures include number of offences that led to conviction 12 months post-randomization and number of emergency room contacts 12 months post-randomization. Data on offences and hospitalizations will be collected by uploading the individual data from patients in the trial to a secure server held by Statistics Denmark and linking data with criminal justice data from the Danish Central Crime Register and hospital data from the Danish National Patient Register approximately 24 months after the conclusion of the trial, in order to allow all data to be fully updated.

### Acceptability and feasibility

Acceptability and feasibility will be measured in two ways: first, participants will complete single-item rating scales at follow-up concerning the helpfulness of their counselling sessions [34]. Clinicians will complete similar the Feasibility of Intervention Measure and the Acceptability of Intervention Measure after the completion of their first two sessions, and after the completion of their first two patients [35].

### Case-control

For the case-control study, controls will be identified through the Danish Register of Drug Users in Treatment and will be selected from among people admitted to treatment during the same period as the cases. Controls will be matched to cases on opioid use, criminal history, psychiatric history, age, sex, duration of present treatment, and previous treatment history.

### Sample size

Sample size was calculated based on the only existing trial [23]. For the primary outcome, retention in treatment, the hazard ratio for the group of patients randomized to ILC compared with treatment as usual was 0.62. We obtained a total sample size of 137 with a power of 80% and an alpha-level of 0.05. We conducted a post hoc power analysis for secondary outcomes (self-reported offending, self-reported substance use). For these measures, we set α = 0.01 due to multiple hypothesis testing and assessed power with three waves of measurement (baseline, three and six months post-randomization) and assumed a correlation of 0.5 between repeated measures. The resulting Cohen’s f that could be measured with a power of 0.95 was 0.16, corresponding to a small to moderate effect size. Finally, for number of offences, we calculated power based on a Poisson distribution; assuming a base-rate of 0.85 offences, the study will have 95% power to measure an incremental risk ratio of 1.43.

### Randomization

Randomization will be performed by means of the minimization method using Minim randomization software, which is a biased-coin approach with a probability of 0.7 to 0.8 for allocation of the “best fitting” treatment [36]. The minimization method obtains an overall balanced distribution of participants, as the number of expected participants is too small for true randomization. Furthermore, participants will be randomized at each site in order to obtain an equal distribution within each municipality/treatment center. This approach was chosen to (a) reduce the impact of geographical variation between the treatment conditions and (b) reduce the risk of counselors not being able to conduct the treatment type to which they were allocated [37]. The following variables will be used for randomization, all based on patient report:

- sex
- age (dummy coded as 0 if age < 40 years, 1 if age ≥ 40 years).
- duration of current OAT treatment (four categories: 0–3 months, 4–5 months, 6–12 months, ≥12 months).
- days of injection drug use in the past month (dummy coded as 0 if 0–9 days, 1 if ≥10).
- having been employed or studying at least three of the past 6 months.
- having received a psychiatric diagnosis from a psychiatrist.

After entering the data for an anonymous new patient into a central database, the referring clinician will contact the Center for Alcohol and Drug Research on a telephone number devised for this purpose and will receive a response to randomization.

At the first counselling session after study enrollment, all participants will be assessed using the YouthMap assessment form [38] or the AdultMap assessment form, depending on the age of the participant. The YouthMap and AdultMap assessment forms are comprehensive assessment tools tailored for people in treatment for psychoactive substance use disorders. The AdultMap is almost identical to the YouthMap, with the exception of a few additional items related to any children in the respondent’s care, as well as fewer items related to education. The follow-up assessments will be conducted via telephone interviews at three and nine months post-randomization. The time plan for the study is shown in Table 1 in Appendix.

#### Data management

Quantitative data will be entered into a secure server located in Denmark and run by SurveyXact. The Centre for Alcohol and Drug Research, Aarhus University will design, set up, and host the database. At the end of the trial, all data will be uploaded to a server at Statistics Denmark in order to allow merging with national registers.

#### Statistical analyses

**Fig. 1:**
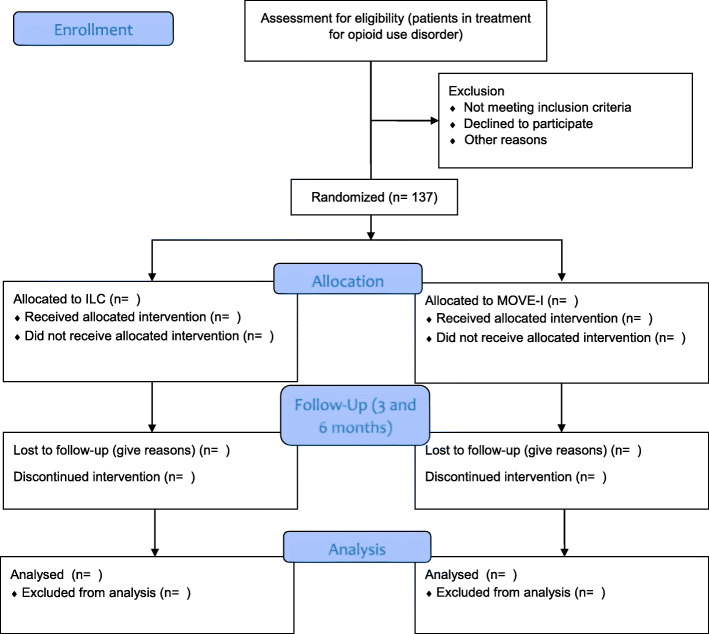
CONSORT Flowchart

Quantitative data analyses will be performed in STATA V16.0. All descriptive analyses, including the recruitment rate, dropouts, losses to follow-up, and the prevalence of serious adverse events will be reported post-randomization and summarized by treatment arm. All causes of withdrawal from randomized treatment will be reported. All of this information will be provided in the form of a CONSORT flowchart (see Fig. 1).

Summaries will be presented as means and standard deviations for variables that are approximately normally distributed, or as medians and interquartile ranges for skewed variables. Categorical variables will be summarized as frequencies and percentages. Transformations will be used when distributional assumptions are not fulfilled for inferential tests on a continuous measure. We will examine and account for the influence of clustering at the site level on the outcomes. All models will adjust for stratification factors and randomized treatment.

The primary analysis will be an intention-to-treat analysis, utilizing all available data from all randomized participants. Retention in OAT will be compared between the two conditions, controlling for sex, age, and time in treatment, using Cox proportional hazards regression. All randomized participants will be analyzed within the treatment arm to which they were originally allocated after randomization, regardless of whether they retained that specific treatment over the course of the trial or not.

Random intercepts will be estimated for site using the shared option in Stata 16. The primary outcome will be analyzed 365 days after the last patient has been randomized, in order to allow sufficient time for dropouts to occur. Secondary outcome analyses will be performed similarly, controlling for baseline values. For sessions attended, a generalized structural equation model will be estimated specifying a Poisson outcome. For severity of drug use, we will estimate a linear regression model with random intercepts for individual patients and sites. This model will control for age and sex and include the interaction between randomization status (ILC versus standard) and assessment wave (baseline, three, or six months).

For number of convictions, we will assess a Poisson, negative binomial, or Poisson or binomial analysis with zero-inflation. The best-fitting model will be selected based on the Bayesian information criterion [39], without regard to the coefficients or which model favors ILC. We will explore the impact of non-engagement with the intervention and loss to follow-up for secondary variables by undertaking exploratory complier adjusted analyses [40]. No interim analyses or formal subgroup analyses are planned.

## Discussion

This trial will be only the second to evaluate the efficacy of a novel psychoeducational program for externalizing behavior problems, ILC. This trial will assess the impact of the program on retention in treatment, severity of drug use, and days of criminal offending in patients with externalizing problems undergoing OAT.

By providing participants with vouchers for attendance, we maximize the likelihood that they will attend sessions regardless of randomization assignment, thus reducing the risk that lack of compliance will mask or exaggerate any real differences between the two conditions. Further, by having a manualized, evidence-based comparison for ILC, we set the standard for showing efficacy higher than in the previous trial.

If ILC turns out to be superior to MOVE-I, it will have strong implications for patients in OAT with externalizing behavior problems. ILC has been shown to be very easy to learn and implement, and no special training is required for clinicians with some experience working with patients with substance use disorders.

Certain limitations are unavoidable, even though they are already known at this point. The influx of new patients into the sites is limited, so it is not possible to include only new admissions.

A further limitation is that the total number of patients is unlikely to be high enough for analysis of moderators of outcome.

## Data Availability

At the conclusion of the study, data will be made available to other researchers at the aggregate level to avoid the sharing of data on individual patients.

## Appendix

**Table 2:** Time plan for the study

	STUDY PERIOD							
	Enrollment	Allocation	Post-allocation			Close-out	Follow-up	
**TIME POINT**	***-t***_***1***_	**0**	***Session 1–2***	***Sessions 3–7***	***Sessions 8–14***	***t***_***x***_	***3 months***	***6 months***
**ENROLLMENT:**								
**Eligibility screen**	X							
**Informed consent**	X							
***No other procedures***	X							
**Allocation**		X						
**INTERVENTIONS:**								
***CBT/MI with voucher reinforcement for attendance***			Assessment	Treatment planning	Work on high-risk situations for drug use	Continued opioid agonist treatment	X	X
***Impulsive lifestyle counselling with voucher reinforcement for attendance***			Assessment	Treatment planning	Impulsive Lifestyle Counselling	Continued opioid agonist treatment	X	X
**ASSESSMENTS:**								
	YouthMap							
***Outcome variables***			Current well-being and substance use (at each session)	Current well-being and substance use	Current well-being and substance use	Retention in treatment	Substance use, treatment, criminal behavior	Substance use, treatment, criminal behavior
***Other variables***			Attendance	Attendance	Attendance			

## Abbreviations

ILC: Impulsive Livestyle Counselling
MOVE-I: Motivational interviewing and cognitive behavioral therapy with vouchers and reminders
OAT: Opioid agonist treatment
OUD: Opioid use disorder
